# Peritoneal tunnels: A site at risk for treatment failure when performing treatments for peritoneal metastases. A case series of 2 patients

**DOI:** 10.1016/j.ijscr.2019.07.026

**Published:** 2019-07-19

**Authors:** Paul H. Sugarbaker

**Affiliations:** Program in Peritoneal Surface Malignancies, MedStar Washington Hospital Center, 106 Irving St., NW, Suite 3900, Washington, DC 20010, USA

**Keywords:** Inguinal canal, Hepatic bridge, Pseudomyxoma peritonei, Reoperative surgery, HIPEC, Residual disease, Cytoreductive surgery

## Abstract

**Background:**

Peritoneal metastases from gastrointestinal cancer can be treated by a combination of surgery to remove visible evidence of disease and perioperative intraperitoneal chemotherapy to eradicate microscopic disease. Complete cytoreduction is necessary for long-term benefit.

**Methods:**

Two sites for incomplete cytoreduction because tumor is hidden from visual inspection were identified. Patients who developed recurrence at these anatomic sites were studied.

**Results:**

A peritoneal tunnel along a patent processus vaginalis allowed tumor to be sequestered along the inguinal canal in a female patient. A peritoneal tunnel beneath a hepatic bridge over the round ligament obscured mucinous tumor in a male patient.

**Conclusions:**

Peritoneal tunnels in the inguinal region or at the porta hepatis should be opened by surgical dissection so that all foci of peritoneal metastases are removed at a complete cytoreductive surgery and these sites treated with chemotherapy.

## Highlights

• For long-term benefit to result from cytoreductive surgery, all visible disease must be removed.
• Peritoneal metastases obscured from visible detection by the surgeon will not be resected.
• Peritoneal tunnels in the inguinal and porta hepatis regions were identified.
• For optimal cytoreduction, peritoneal tunnels must be opened and tumor resected.

## 1 Introduction

The surgical management of peritoneal metastases from gastrointestinal and gynecologic malignancy is a recent innovation that is now pursued worldwide with enthusiasm [1]. The foremost requirement for success is, as is true for all abdominal and pelvic malignancy, completer visible clearance of the neoplastic process. This is referred to in the peritoneal surface malignancy literature as a complete cytoreduction [2]. In a majority of patients with peritoneal metastases this requires both peritonectomy procedures and visceral resections [3,4]. The cytoreductive surgery, which achieves clearance of all visible evidence of cancers, is usually combined with perioperative intraperitoneal chemotherapy, to eradicate microscopic disease [5]. However, all peritoneal surface oncology teams agree that tumor deposits that remain after the cytoreductive surgery will inevitably progress and result in recurrent disease.

There are some structures within the abdomen and pelvis that may be at particularly high risk for persistence and then progression of peritoneal metastases. These sites of recurrence may be observed in patients with low grade mucinous appendiceal neoplasms because the bulk of disease is consistently eradicated by cytoreductive surgery and perioperative chemotherapy [6]. This allows isolated sites of treatment failure to be clearly demonstrated. A structure within the peritoneal space that may, with some repetition, be associated with recurrence is the peritoneal tunnel. Two peritoneal tunnels may exist in patients with peritoneal metastases, the inguinal canal and the hepatic bridge. The manuscript presents patients with persistent and progressive mucinous appendiceal malignancy within the inguinal canal and beneath the hepatic bridge. Surgical precautions to prevent these sites of recurrence are discussed.

Data on these 2 patients was prospectively recorded and then retrospectively reviewed at an academic institution. This research work has been reported in line with the PROCESS criteria [7]. This study was registered as a case series on the www.researchregistry.com website with UIN 4783.

## 2 Patient 1

In September 2013, a 59 year old woman consulted her family physician with abdominal distention increasing over 2 months. Ultrasound of the abdomen showed bilateral ovarian tumors and copious ascites. In October, an exploratory laparotomy was performed by a gynecologic oncologist who performed a hysterectomy and bilateral oophorectomy, omentectomy, appendectomy, and tumor debulking. All specimens showed well-differentiated mucinous adenocarcinoma with the primary site within the appendix. The patient recovered well following surgery and underwent follow-up using CT.

In 2015, CT showed progression of disease and the patient was evaluated by a peritoneal surface oncology team. CEA, normal postoperatively in 2013, had increased to 11. CT showed mucinous tumor widely distributed on parietal peritoneal surfaces especially beneath the right hemidiaphragm and in the pelvis. A cytoreductive surgery lasting 7 h was performed. The procedure required right upper quadrant peritonectomy, greater and lesser omentectomy, cholecystectomy (for cholelithiasis), and pelvic peritonectomy. Hyperthermic intraperitoneal chemotherapy (HIPEC) and early postoperative intraperitoneal chemotherapy (EPIC) were used [4,5]. All specimens were compatible with diffuse peritoneal adenomucinosis (DPAM) [8]. The patient was followed by abdominal and pelvic CT every 6 months for 3 years and then yearly.

**Fig. 1:**
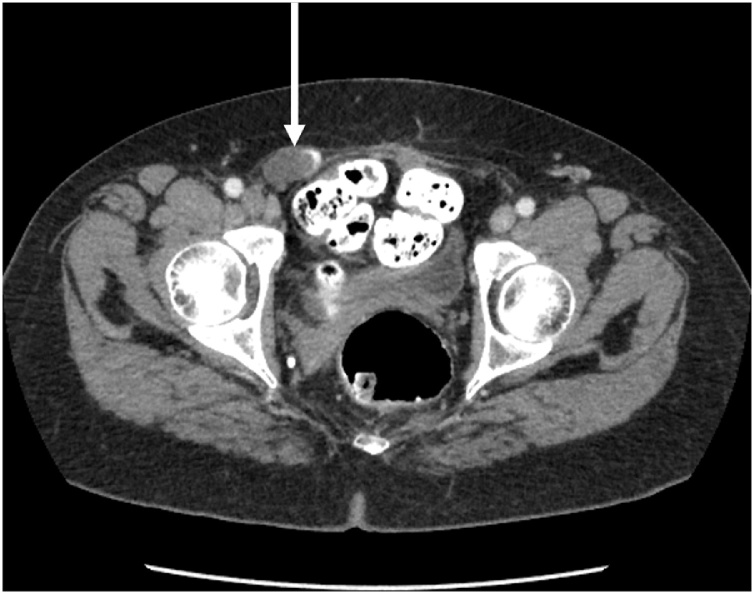
CT cut through the pelvis show a 4 cm cystic mass with peripheral calcification in the right inguinal region. This was compatible with recurrence of pseudomyxoma peritonei 6 years after initial treatment.

In 2019, CT of abdomen and pelvis showed a 4 cm cystic collection in the right inguinal canal (Fig. 1). Also, a 5 cm in greatest diameter cystic mass was associated with the lower pole of the spleen. The patient was asymptomatic. A repeat surgical intervention was recommended. The patient will require a repeat dissection of the inguinal canal (9).

## 3 Patient 2

A 47 year old male noted abdominal distention. A CT and ultrasound of the abdomen led to a diagnosis of pseudomyxoma peritonei. In 2012, the patient underwent a debulking procedure which included peritonectomy, splenectomy, greater omentectomy and appendectomy. HIPEC with cisplatin was performed. Pathology showed well-differentiated peritoneal mucinous adenocarcinoma (PMCA) [8]. The patient was placed in follow-up by CT.

**Fig. 2:**
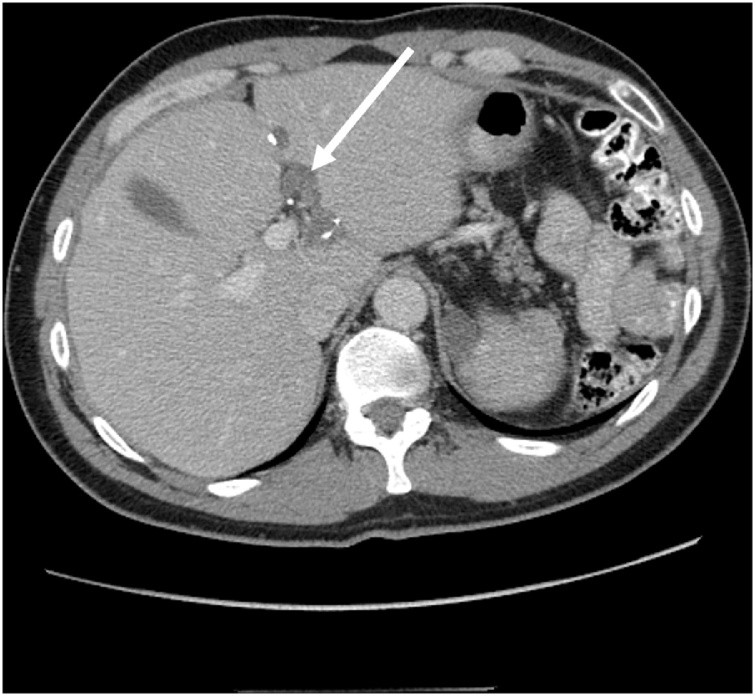
CT cut through the upper abdomen shows cystic collections of mucinous tumor with calcifications along the round ligament. This was compatible with recurrence of pseudomyxoma peritonei beneath a hepatic bridge. This was confirmed at the time of reoperative surgery.

In 2016, CT showed progression of disease. In 2018, CT showed multiple sites of disease progression beneath right and left hemidiaphragm, lesser curvature of stomach and porta hepatis, within the full extent of the prior abdominal incision, and several masses associated with small bowel mesentery. There was a tumor mass filling the rectovesical space. The tumors within the porta hepatis were located along the round ligament (Fig. 2). Segment 3 of the liver was contiguous with segment 4b over the cystic tumor and round ligament. Tumor progression was within the tunnel created by a hepatic bridge.

The patient underwent a 13 -h reoperative cytoreductive surgery that included extensive resection of the abdominal wall along the old abdominal incision, bilateral peritonectomy of the right and left hemidiaphragm, hemigastrectomy with gastrojejunostomy and pelvic peritonectomy.

To resect disease in the porta hepatis, a cholecystectomy was first performed. Then with traction on the left lateral segment of the liver tumor along the fissure defined by the ductus venosus was visualized and resected. There was a tunnel formed by the hepatic bridge. It was type 3 with segment 3 of the liver totally fused over both the tumor and round ligament to liver segment 4b [10]. Using electrosurgery the hepatic bridge was opened. Under direct vision the round ligament was suture ligated and divided as it entered the liver parenchyma. The minimally invasive tumor nodules were dissected away from the liver vasculature without incident. HIPEC was performed prior to gastrointestinal reconstruction and abdominal closure [5].

## 4 Discussion

### 4.1 Peritoneal tunnel in the inguinal canal

The inguinal canal is an abnormal persistence of the processus vaginalis, a peritoneal tunnel that terminates in the scrotal sac in the male or near the labia majora in the female. It contains the spermatic cord in the male and the round ligament in the female [11]. If the inguinal canal remains patent in the female it is referred to as the canal of Nuck. This peritoneal tunnel may give rise to an inguinal hernia or a hydrocele. In our patient 1, a patent processus vaginalis in this 59 year old woman allowed an accumulation of mucinous ascites that contained tumor cells trapped at that site.

The inguinal canal may remain at least partially patent in 20% of adolescent or adult males. It is patent in only a few females. The open peritoneal tunnel in males accounts for the 25% incidence of hernia as the first sign of pseudomyxoma peritonei in males. It leads to a diagnosis of pseudomyxoma peritonei in only 4% of women [12]. If mucoid fluid is recovered from a hernia sac at the time of hernia repair it must be carefully analyzed. If the fluid is mucoid the diagnosis of pseudomyxoma needs to be pursued [13].

The clinical course of patient 1 indicates that a peritonectomy of the pelvis must include an extraction of peritoneum from the inguinal canal. This removal of a potentially patent inguinal canal is more important in the male patient than female patient but can be accomplished in both without damage to cord structures or ilioinguinal nerve. Sugarbaker reported on 17 of 178 patients having new onset or previously repaired inguinal hernias in patients having cytoreductive surgery with HIPEC for pseudomyxoma peritonei. All peritoneal and/or scar from prior hernia repair was resected from the inguinal canal. No repair of the open inguinal canal, which was washed by the chemotherapy solution was attempted. Surprisingly, no recurrent inguinal hernias occurred. Apparently extraction of tumor, peritoneum and scar tissue from the inguinal canal facilitates fibrous closure of the hernia defect so that hernia recurrence was not observed [9].

### 4.2 Peritoneal tunnel at the hilum of the liver

The first mention of a surgically important tunnel at the hilum of the liver was published in 2009 [14]. Sugarbaker described a bridge of liver parenchyma covering the round ligament and documented that peritoneal metastases could be hidden within this tunnel. This small but significant anatomic site of incomplete cytoreduction was thought to cause an isolated progression of peritoneal disease within the porta hepatis. Seven pseudomyxoma peritonei patients who had this unusual manifestation of surgical treatment failure had been reported in 2008 [15]. This was 5% of a total of 140 patients with disease recurrence after cytoreductive surgery and perioperative chemotherapy. Disease persistence in the peritoneal tunnel created around the round ligament and beneath the hepatic bridge was, in retrospect, thought to explain the disease progression in these 7 patients. A cystic tumor accumulation deep within the hilum of the liver was shown by CT. Increasing obstruction of the common or left hepatic duct and progressive atrophy of the left lateral segments of the liver were documented by CT. In 2 patients the greater accuracy of MRI was necessary to document the mucinous tumor extending along the ductal and vascular structures at the porta hepatis [10]. Only 2 of the 7 patients were able to have this focus of disease resected. From this experience an early reoperative intervention of recurrence beneath the hepatic bridge, as occurred in our patient number 2 is recommended. However, now that cytoreductive surgeons know that a peritoneal tunnel often exists and that peritoneal metastases may be hidden from view at this site, the hepatic bridge should be opened and this peritoneal tunnel thoroughly inspected in all patients with peritoneal metastases [14].

A definite description of this anatomic structure, the hepatic bridge, was provided in 2018. In 102 patients undergoing cytoreductive surgery, 50 had a bridge of liver parenchyma over the round ligament between liver segments 3 and 4b creating a peritoneal tunnel. In 52 patients the round ligament could be completely visualized from its origin in the falciform ligament to its entrance into the liver parenchyma. In 44% of patients with a hepatic bridge up to 1/3 of the round ligament was within the peritoneal tunnel (Class 1), in 32% 1/3 to 2/3 of the round ligament was obscured (Class 2), and in 24% more than 2/3 of the round ligament was observed by overlying liver parenchyma (Class 3). In our patient number 2, a class 3 hepatic bridge was present. The electrosurgical division of the liver parenchyma was a full 6 cm long and 3 cm deep [10].

In conclusion, two peritoneal tunnels within the abdominal-pelvic space were identified. These cylindrical spaces may be seeded by peritoneal metastases. Unless the surgeon is aware of peritoneal tunnels as a site of treatment failure, these anatomic sites may be recognized in follow-up as sites of recurrent disease. The inguinal canal is a peritoneal tunnel that may develop progressive disease [13]. Extraction of the peritoneum within the inguinal canal is advised as part of the pelvic peritonectomy procedure. The hepatic bridge covers the round ligament as a result of fusion of liver segments 3 and 4b [14]. If the hepatic bridge is opened as part of a cytoreductive surgical procedure, peritoneal metastases within this peritoneal tunnel can be removed. Knowledge that peritoneal tunnels exist and that peritoneal metastases may persist within is an important part of the surgical management of peritoneal surface malignancy.

## Sources of funding

Data management and secretarial support provided by Foundation for Applied Research in Gastrointestinal Oncology.

## Ethical approval

Local IRB-approval for this case report was not required:

MedStar Health Institutional Review Board has determined that a case report of less than three (3) patients **does not meet the DHHS definition of research** (45 CFR 46.102(d)(pre-2018)/45 CFR 46.102(l)(1/19/2017)) **or the FDA definition of clinical investigation** (21 CFR 46.102(c)) and therefore are not subject to IRB review requirements and **do not require IRB approval**.

This case report is of 2 patients.

## Consent

Written and signed consent was obtained from the patients.

## Author contribution

Paul H. Sugarbaker, MD: study concept or design, data collection, data analysis or interpretation, writing the paper.

## Registration of research

This study was registered as a case series on the www.researchregistry.com website with UIN 4783.

## Guarantor

Paul H. Sugarbaker, MD.

## Provenance and peer review

Not commissioned, externally peer-reviewed.

## Declaration of Competing Interest

Paul H. Sugarbaker has no conflicts of interest to declare.
